# Antibacterial Modes of Herbal Flavonoids Combat Resistant Bacteria

**DOI:** 10.3389/fphar.2022.873374

**Published:** 2022-06-27

**Authors:** Lianyu Song, Xin Hu, Xiaomin Ren, Jing Liu, Xiaoye Liu

**Affiliations:** ^1^ Beijing Traditional Chinese Veterinary Engineering Center and Beijing Key Laboratory of Traditional Chinese Veterinary Medicine, Beijing University of Agriculture, Changping, China; ^2^ Animal Science and Technology College, Beijing University of Agriculture, Changping, China

**Keywords:** natural plant flavonoids, heat-clearing Chinese medicine, antibiotic resistance, multidrug resistant bacteria, antibacterial modes

## Abstract

The increasing dissemination of multidrug resistant (MDR) bacterial infections endangers global public health. How to develop effective antibacterial agents against resistant bacteria is becoming one of the most urgent demands to solve the drug resistance crisis. Traditional Chinese medicine (TCM) with multi-target antibacterial actions are emerging as an effective way to combat the antibacterial resistance. Based on the innovative concept of organic wholeness and syndrome differentiation, TCM use in antibacterial therapies is encouraging. Herein, advances on flavonoid compounds of heat-clearing Chinese medicine exhibit their potential for the therapy of resistant bacteria. In this review, we focus on the antibacterial modes of herbal flavonoids. Additionally, we overview the targets of flavonoid compounds and divide them into direct-acting antibacterial compounds (DACs) and host-acting antibacterial compounds (HACs) based on their modes of action. We also discuss the associated functional groups of flavonoid compounds and highlight recent pharmacological activities against diverse resistant bacteria to provide the candidate drugs for the clinical infection.

## Introduction

**FIGURE 1:**
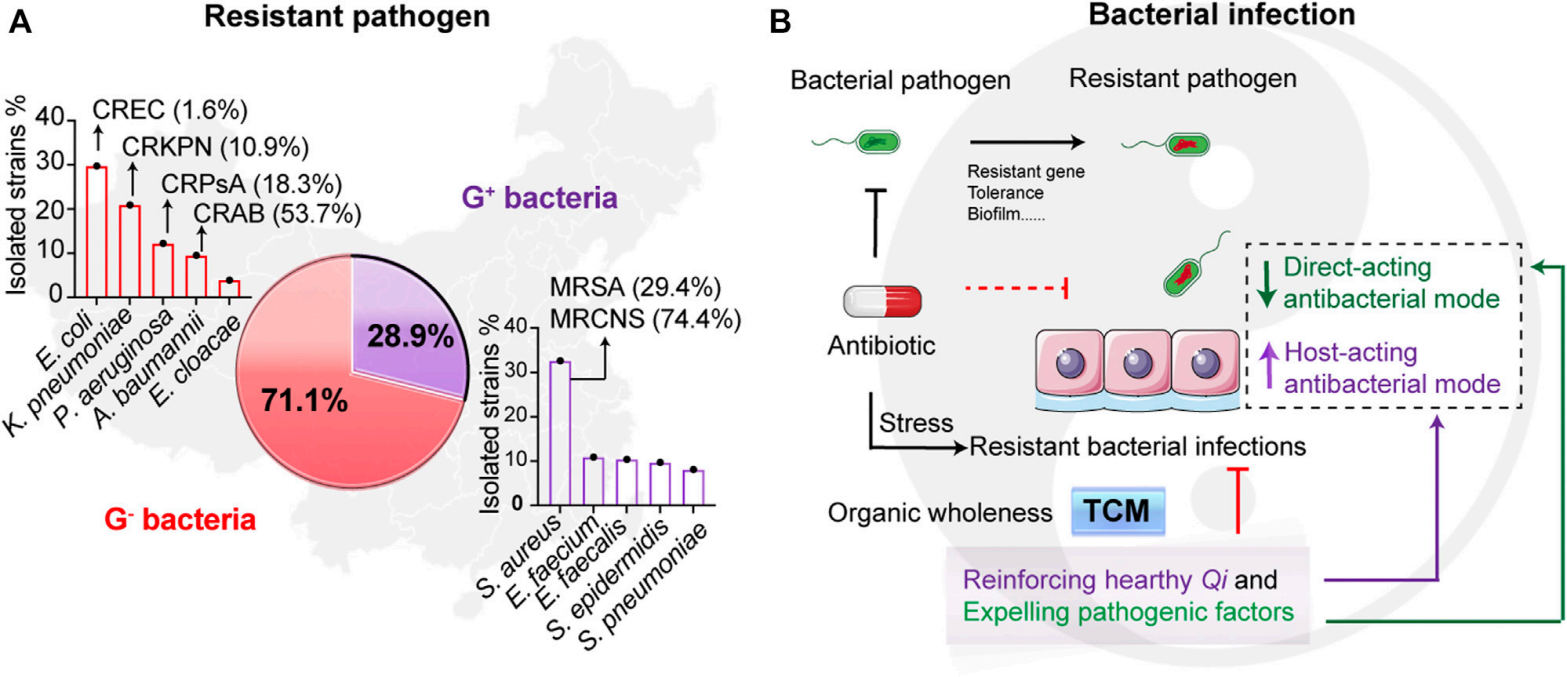
Resistant pathogenic infections and the treatment of traditional Chinese medicine. **(A)** The occurrence of resistant pathogens in China from 10/2019 to 12/2020. Reported data are collected from China Antimicrobial Resistance Surveillance System (CARSS)^1^. The isolated resistant strains from the report that account for Gram-negative (G^−^) bacteria and Gram-positive (G^+^) bacteria are 71.1% and 28.9%, respectively. **(B)** Scheme of the infectious therapy of traditional Chinese medicine (TCM). Resistant bacterial infections lead to the treatment failures of antibiotics. While therapeutic principles of TCM focus on the organic wholeness referring to “reinforcing healthy *Qi* and expelling pathogenic factors,” which displays that both suppressing bacteria and enhancing host defense. The TCM can divide to two types including direct-acting antibacterial mode and host-acting antibacterial mode. It is worth noting that “*Qi*” in TCM denoted the gas of host healthy, mostly meaning the forces of host defense.

The worldwide spreading of pathogenic resistant bacteria threatens public health. Currently, the infections caused by Gram-negative (G^−^) bacteria occur more frequently than Gram-positive (G^+^) bacteria in clinics. A report from the China Antimicrobial Resistance Surveillance System (CARSS)^1^ shows that G^−^ bacteria accounted for 71.1% of the 3,249,123 clinical isolated strains, while Gram-positive (G^+^) bacteria for 28.9% (Figure 1A). Among them, the high number of multidrug resistant (MDR) bacterial infections, such as carbapenem-resistant G^−^ bacteria (CRGNB), are life-threating (Bassetti et al., 2021; Palacios-Baena et al., 2021). As shown in Figure 1A, many more MDR pathogens are emerging, including the resistant G^−^ bacteria like carbapenem-resistant *E. coli* (CREC, 1.6%), carbapenem-resistant *K. pneumoniae* (CRKPN, 10.9%), carbapenem-resistant *P. aeruginosa* (CRPsA, 18.3%), carbapenem-resistant *A. baumannii* (CRAB, 53.7%), and the resistant G^+^ bacteria such as methicillin-resistant *S. aureus* (MRSA, 29.4%) and methicillin-resistant coagulase-negative *S. aureus* (MRCNS, 74.4%). Worse still, global patient deaths due to antibiotic resistance are approximately 700, 000 and the numbers are expected to increase to 10 million by 2050 if no effective measures are introduced^2^. Therefore, alternative strategies to antibiotics and the discovery of novel antibacterial drugs to combat resistant bacteria are in high demand.

In the resistant era, the major therapy of MDR still rely the efficacy and safety antibacterial agents, while the discovery and development of antibacterial drugs are barrier by the unknown infective route of pathogen (Liu et al., 2019; Song et al., 2020; Zulauf and Kirby, 2020). Effective antibacterial therapies need to explore more antibacterial compounds and their pharmacological activities and targets to sustainably combat the resistant bacteria (Theuretzbacher et al., 2020). Our previous work has demonstrated that the host defenses are critical for both bacterial infections and antibacterial drug therapy (Liu F. et al., 2020; Liu et al., 2021). Therefore, host factors are vital for the management of MDR bacterial infections. In most situations, bacteria evolve or develop a variety of strategies to infect the human host (Culyba and Van Tyne, 2021). Thus, antibiotics cannot effectively fight against various bacterial mutations, while large doses and frequent usage of antibiotics can cause bacteria to be constantly exposed to drug stress, which will trigger the emergence of drug-resistant bacteria.

Unlike the direct stress of antibiotics to bacteria, host-directed therapy (HDT) is a sensible strategy against unknown resistant bacteria, and host-acting antibacterial compounds (HACs) are worthy of developing (Liu et al., 2022). The intrinsic advantages of HACs therapy are mobilizing the host cells to protect themselves from unknown infections with less emergence of resistant bacteria, due to the reduced selective pressure from directly targeting bacteria. Similar to HDT and HACs, the main therapeutic principle of traditional Chinese medicine (TCM) against bacteria is dependent on “reinforcing healthy *Qi* and expelling pathogenic factors,” which also refers to its abilities of both enhancing host defense forces and eliminating pathogenic bacteria (Figure 1B). In terms of the TCM theory, we divide the antibacterial actions of TCM to direct-acting and host-acting antibacterial modes (Figure 1B). Upon infections, bacterial diseases belong to heat syndrome (Wang et al., 2018). Heat-clearing Chinese medicines symptomatically treat bacteria-caused internal heat syndrome. Therefore, the heat-clearing herbs may serve as potential drug libraries for screening the lead compounds to combat MDR bacterial infection. Flavonoids are abundant in plants, such as in most herbs, and recent research shows that flavonoids have excellent antibacterial activities that are regulated tightly with their functional groups (Song et al., 2021a).

Overall, this review focuses on the flavonoids from heat-clearing herbs to reveal the therapeutic strategies of resistant pathogens. In the following sections, we discuss the availabilities and antibacterial pipelines of herbal flavonoids.

## Herbal Flavonoids in Heat-Clearing Medicines

**FIGURE 2:**
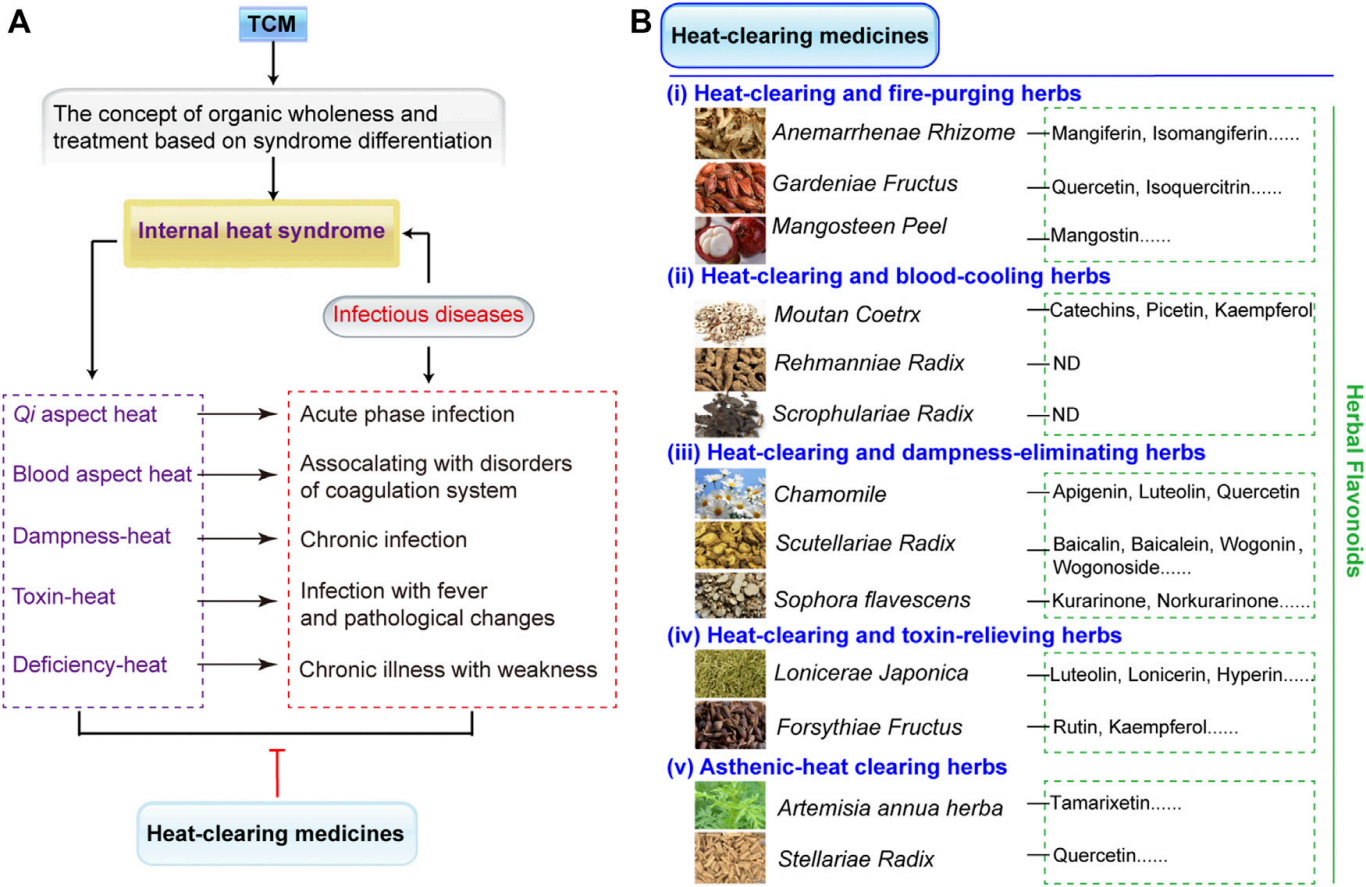
Heat-clearing medicines and the distribution of its flavonoid compounds. **(A)** Heat-clearing medicines treat internal heat syndrome. TCM is identified by the concept of organic wholeness and treatment based on syndrome differentiation. The syndrome differentiation of infectious diseases belongs to internal heat syndrome, which can be treated with heat-clearing medicines. Internal heat syndrome contains *Qi* aspect heat, blood aspect heat, dampness-heat, toxin-heat, and deficiency-heat and occur during the spatiotemporal axis of infectious diseases, such as acute phase, disorders of coagulation system, chronic infection, fever, pathological changes, and chronic illness with weakness. **(B)** The classifications of heat-clearing medicines that combat different heat syndromes include (i) heat-clearing and fire-purging herbs, (ii) heat-clearing and blood-cooling herbs, (iii) heat-clearing and damp-eliminating herbs, (iv) heat-clearing and toxin-relieving herbs, and (v) asthenic-heat clearing herbs. In addition, the heat-clearing herbs that contain herbal flavonoids are listed. ND denotes no detected flavonoids in heat-clearing herbs. All the species list in the figure are fully validated^3^.

TCM is identified by organic wholeness and treatment based on syndrome differentiation. The syndrome differentiation of infectious diseases belongs to internal heat syndrome, which can be treated with heat-clearing medicines. Internal heat syndrome manifests itself in many forms including *Qi* aspect heat, blood aspect heat, dampness-heat, toxin-heat, and deficiency-heat (Figure 2A) (Chen, 1965). The heat syndrome occurs in the spatiotemporal axis of infectious diseases’ correspondence with acute phase, disorders of coagulation system, chronic infection, fever, pathological changes, and chronic illness with weakness (Figure 2A). For these heat syndromes, heat-clearing medicines have five of therapy: 1) heat-clearing and fire-purging herbs, 2) heat-clearing and blood-cooling herbs, 3) heat-clearing and damp-eliminating herbs, 4) heat-clearing and toxin-relieving herbs, and 5) asthenic-heat clearing herbs (Figure 2B).

Flavonoids are widely distributed heat-clearing medicines and exhibit multiple biological activities such as antibacterial, anti-inflammatory, and antioxidation effects (Lan et al., 2021; Waditzer and Bucar, 2021). The chemical structures of more than 8000 flavanoids have been determined (Wen et al., 2021) and most existing flavonoids have been reported to defend against MDR bacterial infections (Song et al., 2021a). As shown in Figure 2B, the typical heat-clearing and fire-purging herbs include *Anemarrhenae Rhizome*, *Gardeniae Fructus,* and *Mangosteen peel*. Mangiferin and isomangiferin are the main compounds from *Anemarrhenae Rhizome* (Piwowar et al., 2020), quercetin and isoquercitrin can be extracted from fruits of *Gardeniae Fructus* (Kim et al., 2006), and mangostin exsits in the *Mangosteen peel* (Weerayuth et al., 2014). Heat-clearing and blood-cooling herbs contain *Moutan Coetrx*, *Rehmanniae Radix,* and *Scrophulariae Radix*; among these, only *Moutan Coetrx* is reported to contain flavonoid compounds of catechins, picetin, and kaempferol (Zhang et al., 2017), while the others have no detected flavonoids. Three heat-clearing and damp-eliminating herbs with large flavonoids are apigenin, luteolin, andquercetin in *Cancrinia discoidea* (Su et al., 2011), baicalin, baicalein, wogonin, and wogonoside in *Scutellariae Radix* (Guowei et al., 2018), and kurarinone and norkurarinone in *Sophora flavescens* (Dong et al., 2021)*.* Heat-clearing and toxin-relieving herbs such as *Lonicerae Japonica* and *Forsythiae Fructus* have luteolin, lonicerin, hyperin, and rutin (Huang et al., 2020; Wei et al., 2020). Finally, asthenic-heat clearing herbs include *Artemisia annua herba* and *Stellariae Radix* and these exist in tamarixetin and quercetin, correspondingly (Hao et al., 2020).

Antibacterial approaches and the main targets of the flavonoids are important for clinical applications. Within the decoding of active function, the classifications of flavonoids based on the chemical structures are intuitive, simple, and critical for understanding of antibacterial activates of flavonoids.

## Chemical Structure Classification of Flavonoids

**FIGURE 3:**
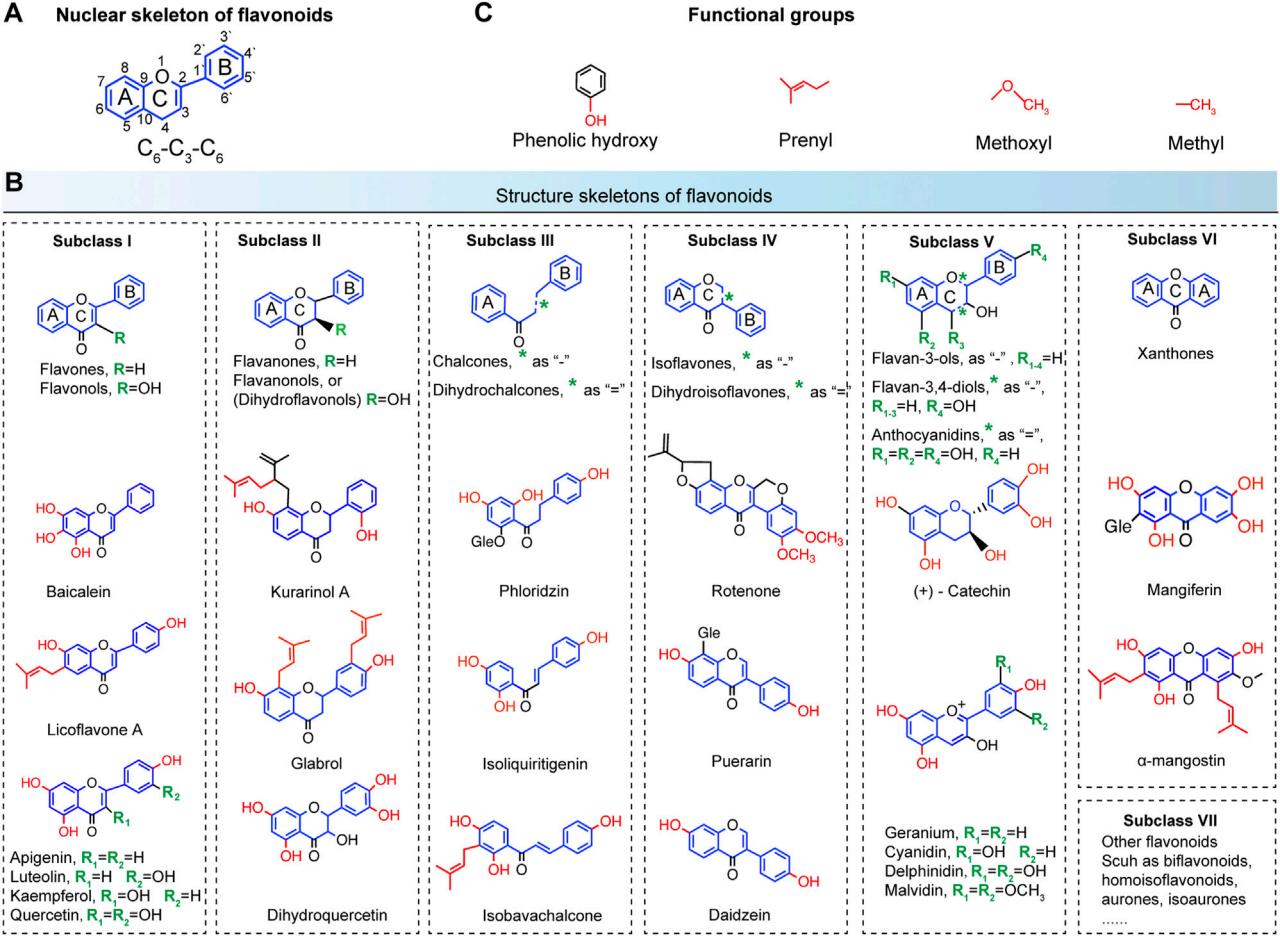
Chemical structure classifications of flavonoids. **(A)** The nuclear skeleton of flavonoids contains a 2-phenyl-chromone core with the C_6_-C_3_-C_6_ system. The A and B benzene rings connect to each other through the central three carbon atoms, which can or cannot form the C ring. **(B)** The major subclasses of flavonoids. Subclass I, flavones and flavonols; Subclass II, flavanones and flavanonols; Subclass III, chalcones and dihydrochalcones; Subclass IV, isoflavones and dihydroisoflavones; Subclass V, flavan-3-ols, flavan-3.4-diols and anthocyanidins; Subclass VI, xathones and mangiferin; Subclass VII, other flavonoids such as bioflavonoids, homoisoflavonoids, aurones, isoaurones, and so on. The common skeletons are marked by blue and the green R groups denote substitutable groups **(C)** The main functional groups of flavonoids include phenolic hydroxy, prenyl, methoxyl, and methyl (highlighted in red).

To assess the antibacterial activities of flavonoids, the chemical structures of flavonoid compounds need to be clarified. Thus, the structural classifications of herbal flavonoids are detailed in Figure 3. Generally, flavonoids refer to a series of compounds in which two benzene rings (A and B rings) with phenolic hydroxyl groups are connected to each other through the central three carbon atoms (Wen et al., 2021). As shown in Figure 3A, the nuclear skeleton of flavonoids forms the C_6_-C_3_-C_6_ system. This subsequently classifies the flavonoids according to the degree of oxidation of the central three carbon atoms, the linking position of the B ring, and whether the three carbon chains constitutes a ring (C ring) or not (Xie et al., 2015). Major subclasses of flavonoids are detailed in Figure 3B; subclass I includes flavones and flavonols, Subclass II includes flavanones and flavanonols, Subclass III includes chalcones and dihydrochalcones, Subclass IV includes isoflavones and dihydroisoflavones, Subclass V includes flavan-3-ols, flavan-3.4-diols, and anthocyanidins, and Subclass VI includes xathones and mangiferin, Subclass VII includes other flavonoids such as bioflavonoids, homoisoflavonoids, aurones, isoaurones, and so on. The common skeletons are marked by blue and the green R groups denote substitutable groups. Finally, the functional groups of flavonoids including prenyl, methoyl, and methyl (highlighted in red) decide the antibacterial activities (Figures 3B,C), resulting in various modes of action on resistant bacteria.

## Modes of Action of Flavonoids on Resistant Bacteria

Antibacterial modes of flavonoids depend on the structures, that is the substitutions on the aromatic rings. As more antibacterial activities of natural flavonoids have been found, numerous flavonoids have been confirmed to have existing antibacterial activity-structure relationships. For instance, the flavonoid compounds that have prenyl groups with hydrophobic substituents inhibit bacterial membrane function and biofilm formation (Rashid et al., 2007; Prawat et al., 2013; Wenzel, 2013; Narendran et al., 2016). The flavonoid compounds that have phenolic hydroxy groups with scavenging oxygen free radicals can modulate antioxidation and anti-inflammatory activities (Wenzel, 2013; Guowei et al., 2018; Farhadi et al., 2019; Jordan et al., 2020).

According to different modes of action, flavonoid compounds can be grouped into two types, one with a direct-acting antibacterial mode (DAC) regulated by prenyl (Dong et al., 2017; Kariu et al., 2017; Jordan et al., 2020; Song et al., 2021a), the other with host-acting antibacterial mode (HAC) regulated by phenolic hydroxy (Xie et al., 2015; Echeverria et al., 2017; Farhadi et al., 2019; Liu et al., 2022).

**FIGURE 4:**
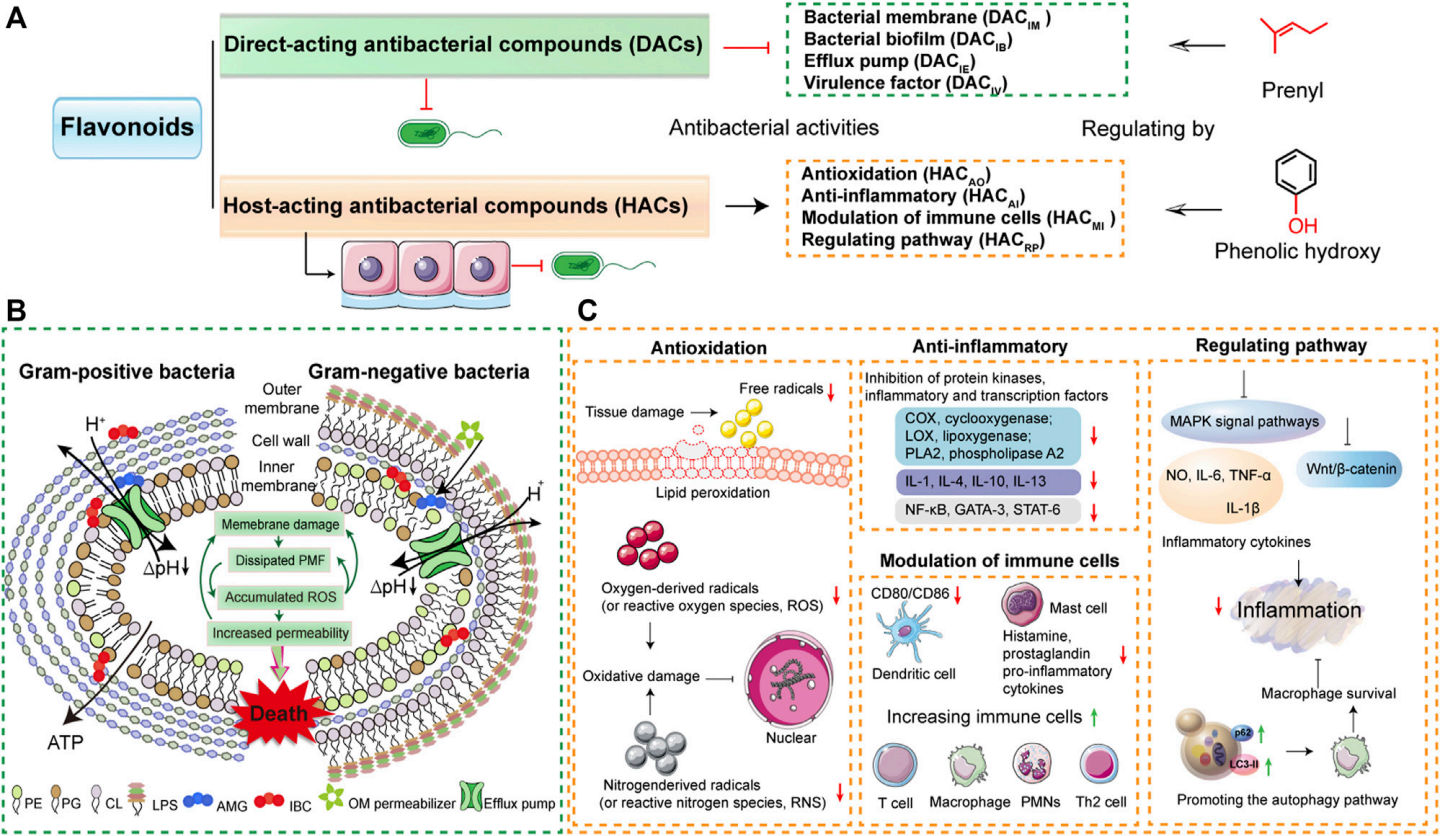
Modes of action of flavonoids on resistant pathogens. **(A)** The flavonoids are divided into two types of compounds based on the antibacterial modes. One is the direct-acting antibacterial flavonoid compounds (DACs) that damage the bacterial membrane and inhibit bacterial biofilm, bacterial efflux pump, or virulence factor. The other refers to host-acting antibacterial flavonoid compounds (HACs) that target anti-inflammatory, antioxidation modulation of immune cells, or regulate cellular pathway. The antibacterial activities of DACs and HACs are regulated by prenyl, phenolic hydroxy, or methyl. **(B)** The mechanisms of flavonoid DAC (isobavachalcone, AMG and α-mangostin, IBC) inhibit bacterial membrane (Song et al., 2021a). The copyright was obtained from Wiley-VCH GmbH in their journal of *Advanced Science* with the terms of the https://creativecommons.org/licenses/by/4.0/license. **(C)** The molecular mechanisms of flavonoid HACs have the antibacterial actions of antioxidation, anti-inflammatory, modulation of immune cells, and regulating cellular pathway.

For a clearer understanding of the two modes, the related three subcategories are attributed to these two modes. According to the antibacterial activities of inhibition of bacterial membrane, bacterial biofilm, efflux pump, and virulence factor, DAC divides to DAC_IM_, DAC_IB_, DAC_IE_, and DAC_IV_, respectively. HAC is divided into HAC_AO_, HAC_AI_, HAC_MI_, and HAC_RP_ based on the effects on the antioxidation, anti-inflammatory, modulation of immune cells, and regulating pathway of host (Figure 4A and Table 1). Furthermore, the specific modes of action on herbal flavonoids that combat resistant bacteria are illustrated in the following.

**TABLE 1:** Antibacterial modes of natural plant flavonoids and the main target bacteria.

Flavonoids	Flavonoid structures	Sources	Antibacterial modes (DAC_IM/IE/IV/IB_/HAC_AO/AI/MI/RP_)	Target	Bacteria	References
Flavonoids	Prenylated flavonoids	*Epimedium* species	DAC_IV/IB_	Bacterial biofilm formation	*Porphyromonas gingivalis*	Kariu et al. (2017)
	Flavonoids	*Sophora flavescens* ^a^	HAC_AI/RP_	Autophagy protein LC3II and p62	Tuberculosis (TB)	Kan et al. (2020)
Flavones	Apigenin	*Cancrinia discoidea* ^a^	DAC_IE_; HAC_AI/AO_	Inhibition of EtBr efflux pump	*Staphylococcus aureus*	(Brown et al., 2015; Tang et al., 2017; Wang et al., 2019; Ciumarnean et al., 2020)
Flavonols	Baicalein, Baicalin	*Scutellariae Radix* ^a^	DAC_IM/IE/IV/IB_; HAC_AI/AO_	Bacterial biofilm formation	MRSA; *S. aureus*; *Streptococcus suis*; *Helicobacter pylori*; *Pseudomonas aeruginosa*	(Rajkumari et al., 2017; Chen et al., 2018; Liu et al., 2020; Zhang et al., 2020; Lu et al., 2021; Palierse et al., 2021; Xiao et al., 2021)
	Baicalein-7-O-β-D-glucuronide	*Scutellariae Radix* ^a^	HAC_AI/AO/RP_	Regulating Wnt/β-catenin and MAPK signal pathways	Bacteria	(Li et al., 2009; Kawai, 2018)
	Quercetin, Isoquercitrin	*Gardeniae Fructus* ^b^; *Stellariae Radix* ^c^	DAC_IM/IE/IV_; HAC_AO_		CRGNB; *S. aureus*; CRPsA; CRAB	(Lee et al., 2017; Mohamed et al., 2020; Pal and Tripathi, 2020; Yang et al., 2020)
	Kaempferol	*Moutan Coetrx* ^d^	DAC_IM/IV_		MRSA, *S. aureus*	(Aparna et al., 2014; Lin et al., 2020; He et al., 2021)
	Luteolin, Lonicerin	*Lonicerae Japonica* ^e^	DAC_IM/IE/IV/IB_; HAC_AI/MI/_	Reducing the extracellular matrix to inhibit microcolony biofilms; MsrA efflux pump	*Escherichia coli; Klebsiella pneumoniae*; MRSA; *Trueperella pyogenes*; *Mycobacterium tuberculosis*	(Zhang et al., 2018; Pruteanu et al., 2020; Miao et al., 2021; Singh et al., 2021; Guo et al., 2022)
	Luteolin-7-O-β-D-glucuronide	*Impatiens balsamina*	HAC_AI/RP_	MAPKs pathway	G^−^ Bacteria	(Liu et al., 2020; Pereita et al., 2020)
	Tamarixetin	*Artemisia annua herba* ^c^	DAC_IV_; HAC_AI_		*Escherichia coli* K1	Park et al. (2018)
	Wogonin	*Scutellariae Radix* ^a^	DAC_IE/IV_	Inhibition of EtBr efflux pump	*S. aureus*, *Mycobacterium aurum* and *Mycobacterium smegmatis*	Solnier et al. (2020)
Flavanones	Glabrol	*Liquorice* (n.a.)	DAC_IM_	LPS	MRSA	Kalli et al. (2021)
Flavanonols	Hyperin, Hyperoside	*Lonicerae Japonica* ^e^; *Anadenanthera colubrina var cebil* (n.a.)	HAC_AO_	Antioxidant potentials	*S. aureus*	Rodrigo Cavalcante de Araújo et al. (2019)
	Kurarinol A, Kurarinone	*Sophora flavescens* ^a^	HAC_MI/AI_	Regulation of macrophage functions	Gram-negative bacteria	Xu et al. (2021)
	Rutin	*Forsthiae Fructus* ^a^	DAC_IB/IV_; HAC_AO_	Bacterial biofilm, atioxidant potentials, inflammatory cytokine expressions	*P. aeruginosa*; *S. aureus*; Multidrug-resistant Gram-positive pathogens; Drug resistant *Aeromonas hydrophila*	(Deepika et al., 2019; Di Lodovico et al., 2020; Motallebi et al., 2020; Suebsaard and Charerntantanakul, 2021)
Chalcones	Phloridzin	Apples, tea (n.a.)	DAC_IB_; HAC_AI_	Efflux protein genes Biofilm formation	*S. aureus* (msrA and norA efflux protein); Gram-negative bacteria	(Lopes et al., 2017; Kamdi et al., 2021)
Dihydrochalcones	Isoliquirtigenin	*Liquorice* (n.a.)	DAC_IM_; HAC_AI_	Bacterial cytoplasmic membrane function, Tissue inflammation	MRSA	(Gaur et al., 2016; Watanabe et al., 2016)
	Isobavachalcone	*Psoralea corylifolia* (n.a.)	DAC_IE_	AcrAB, TolC efflux pumps	MDR-Gram-negative bacteria; MRSA; Bacteria	(Kuete et al., 2010; Song et al., 2021a)
Isoflavones	Rotenone	*Derris* (n.a.)	-	-	-	-
Dihydroisoflavones	Puerarin	*Pueraria Lobata* (n.a.)	HAC_AI_	Anti-inflammation by protecting the epithelia and goblet cells and increasing the short-chain fatty acids level	Pathogenic bacteria	(Wu et al., 2020; Song et al., 2021a)
	Daidzein	n.a.	DAC_IE_	Efflux pump assemblies and AcrB and MexB proteins	*E. coli* (AcrB efflux pump); *P. aeruginosa* (MexB efflux pump)	Aparna et al. (2014)
Flavan-3-OLS Flavan-3,4-Diols	Catechin	*Moutan Coetrx* ^d^; *Hyperforin perforatum* ^e^; *Ginkgo Biloba,* tea (n.a.)	DAC_IM/IV_; HAC_AI_	Inhibition of bacterial membrane and bacterial virulence factors, anti-inflammation	Resistant bacteria	(Gomes et al., 2018; Yang et al., 2018; Siebert et al., 2021; Wu and Brown, 2021)
Anthocyanidins	Cyanidin, Delphinidin, Geranium, Malvidin	Angiosperm (n.a.)	-	-	-	-
Xanthones	Mangiferin	*Anemarrhenae Rhizome* ^b^	DAC_IE_	Efflux pumps	Gram-negative MDR bacteria.	Tchinda et al. (2019)
	α-mangostin	*Mangosteen peel* ^b^	DAC_IM_	PG of bacterial membrane function	Bacteria	Song et al. (2021a)

Note: Heat-clearing Chinese herbs include.

a Heat-clearing and damp-eliminating herbs.

b Heat-clearing and fire-purging herbs.

c Asthenic-heat clearing herbs.

d Heat-clearing and blood-cooling herbs.

e Heat-clearing and toxin-relieving herbs.

n.a., not applicable. DAC represents direct antibacterial flavonoid compound, DAC_IM_, DAC that inhibition of bacterial membrane, DAC_IE_, DAC that inhibition of bacterial efflux pump, DAC_IV,_ DAC that inhibition of bacterial virulence factor, DAC_IB_, DAC that inhibition of bacterial biofilm; HAC denotes host-acting antibacterial flavonoid compound, HAC_AI_, anti-inflammatory of HAC; HAC_MI_, HAC that modulation of immune cells, HAC_AO_, antioxidation of HAC, HAC_RP_, HAC that regulate signaling pathways; MDR, multidrug-resistant; MRSA, Methicillin-resistant *Staphylococcus aureus*; CRGNB, carbapenem-resistant Gram-negative bacteria; CRPsA, carbapenem-resistant *Pseudomonas aeruginosa*; CRAB, carbapenem-resistant *Acinetobacter baumannii*; “-” denotes that not report the antibacterial activity. All the species listed in the table are fully validated^3^.

### Direct-Acting Antibacterial Modes

Direct-acting antibacterial modes of herbal flavonoids means that the antibacterial agents directly target the bacterial themselves, such as the bacterial membrane functions, biofilm formation, efflux pumps, and virulence factors (Figure 4A). Of the activities of DAC flavonoids, the first category is the biophysical barrier of the bacterial inner membrane that directly increases bacteria survival (Martin et al., 2020). Similar to most antibacterial drugs, the main therapeutic treatment option of flavonoids is damaging bacterial membrane functions. A recent report shows that the antibacterial mechanisms of flavonoids rely on distinctive modes of action to bind the phospholipids of bacterial membrane, which result in the disruption of proton motive force and metabolic disturbance (Song et al., 2021a). DAC flavonoids such as isobavachalcone (AMG) and α-mangostin (IBC) target the phosphatidylglycerol (PG) of bacterial membrane (Figure 4B). These two DAC flavonoids have 3′-prenyl and 2, 8-prenyl, respectively (Figure 3B). Active prenyl flavonoids endow the antibacterial activities that combat most bacteria, even MRSA (Table 1). In addition, the prenyl flavonoid compounds are abundantly distributed in herbal flavonoids (Wen et al., 2021). For instance, as typical heat-clearing and damp eliminating herbs, *Scutellariae Radix* has the main flavonoes of baicalein and baicalin using the direct-acting antibacterial activity to fight against MRSA, *S. aureus,* and *Streptococcus suis* infections *via* inhibition of bacterial membrane functions (Rajkumari et al., 2017; Chen et al., 2018; Zhang et al., 2020; Lu et al., 2021) (Table 1). In addition, other flavonoid compounds in heat-clearing herbs also have the antibacterial abilities of damaging the bacterial membrane, such as quercetin from *Gardeniae Fructus* (iii, heat-clearing and damp eliminating herb) (Mohamed et al., 2020; Yang et al., 2020), kaempferol origin from *Moutan Coetrx* (ii, heat-clearing and blood-eliminating herb) (Lin et al., 2020; He et al., 2021), luteolin and lonicerin from *Lonicerae Japonica* (iv, heat-clearing and toxin-relieving herb) (Zhang et al., 2018; Singh et al., 2021), hyperin and hyperoside origin from *Lonicerae Japonica* (iv, heat-clearing and toxin-relieving herb) (Rodrigo Cavalcante de Araújo et al., 2019), rutin from *Forshiae Fructus* (iii, heat-clearing and damp eliminating herb) (Deepika et al., 2019; Di Lodovico et al., 2020; Motallebi et al., 2020), and mangiferin from *Anemarrhenae Rhizome* (i, heat-clearing and blood-cooling herbs) (Tchinda et al., 2019). These DAC_IM_ flavonoids that treat bacterial strains are detailed in Table 1.

In order to gain more survival options from the antibiotic stress, resistant bacteria evolved various approaches to survive, such as biofilm, efflux pumps, and virulence factors (Blair et al., 2015; Narendran et al., 2016; Ercoli et al., 2018; Yelin and Kishony, 2018). Flavonoids limited the spread of resistant bacteria by serving as the inhibitors of bacterial efflux pumps (Solnier et al., 2020) and bacterial virulence factors (Wang et al., 2020). We summarized the effect of flavonoids such as DAC_IB_, DAC_IE,_ and DAC_IV_ in Table 1. For the DAC_IB_ mode, flavonoids directly target the bacterial biofilm formation as therapeutic strategies. For instance, the baicalein-fabricated gold nanoparticles have antibiofilm activity against *Pseudomonas aeruginosa* PAO1 (Rajkumari et al., 2017). The flavone luteolin and the flavonols myricetin, morin, and quercetin strongly reduce the extracellular matrix to interrupt the *Escherichia coli* macrocolony biofilms by directly inhibiting the assembly of amyloid curli fibers by driving CsgA subunits into an off-pathway leading to SDS-insoluble oligomers (Pruteanu et al., 2020). With flavonoid DAC_IE_, apigenin can recover the susceptibility of antibiotics to resistant bacteria by suppressing the EtBr efflux pump (Brown et al., 2015; Tang et al., 2017; Wang et al., 2019; Ciumarnean et al., 2020). Additionally, wogonin can suppress the EtBr efflux pumps of *S. aureus*, *Mycobacterium aurum*, and *Mycobacterium smegmatis* (Solnier et al., 2020). Luteolin inhibits MsrA efflux pump in *Trueperella pyogenes* (Guo et al., 2022), phloridzin inhibits the msrA and norA efflux proteins of *S. aureus* (Lopes et al., 2017; Kamdi et al., 2021), isobavachalcone inhibits AcrAB, TolC efflux pumps of G^−^ bacteria (Kuete et al., 2010), and daidzein inhibits AcrB efflux pump of *E. coli* (Aparna et al., 2014). It is worth noting that quercetin that belongs to flavanols have the inhibitory potential to effectively act as the efflux pump to treat CRNB due to its polyphenol hydroxyl group structure (Pal and Tripathi, 2020). On the other side, flavonoid compounds have the direct-acting antibacterial effect of inhibiting bacterial virulence factors, such as α-hemolysin (Hla) of *S. aureus* (Wang et al., 2020; He et al., 2021). The Hla can cause bacterial entry of host cells and also lead to bacterial coinfection, while flavonoids can target Hla to control bacterial infection (He et al., 2021). Altogether, the flavonoid compounds that inhibit virulence factors are denoted as DAC_IV_ flavonoids and summarized in Table 1.

### Host-Acting Antibacterial Modest

To establish persistent infection in the host, resistant bacteria toned to interrupt the host defense and trigger inflammation (Ercoli et al., 2018; Mathur et al., 2019; Pham et al., 2020; Rowe et al., 2020). The host is the key point to drug therapy and HDT may be a novel approach for controlling resistant bacteria (Kaufmann et al., 2018; Ahmed et al., 2020). Many natural plant compounds exhibited the antibacterial activity of HDT, such as the bacterial infection therapy obtained from bedaquiline from *Perucian bark* that activates host innate immunity (Giraud-Gatineau et al., 2020). Additionally, the host-acting antibacterial compounds were systematically summarized in our previous works (Liu et al., 2022). Based on these foundations, as shown in Figure 4C, we divide HAC flavonoids into four groups: antioxidation (HAC_AO_), anti-inflammatory (HAC_AI_), modulation of immune cells (HAC_MI_), and regulating cellular pathways (HAC_RP_).

HAC_AO_ flavonoids are able to combat bacterial infection with their antioxidation function, by reducing the free radicals and lipid peroxidation to inhibit oxygen-derived radicals or nitrogen-derived radicals to avoid oxidative damage (Kamdi et al., 2021; Palierse et al., 2021) In addition, this antibacterial activity mainly relies on the phenolic hydroxy group of flavonoid compounds, such as quercetin and hyperoside (Figures 3C, 4A) (Xie et al., 2015). For the HAC_AI_ flavonoids, the major anti-inflammatory modes of flavonoid compounds are inhibition of protein kinases (COX, cyclooxygenase, LOX, lipoxygenase and PLA2, phospholipase A2), inflammatory factors (IL-1, IL-4, IL-10, and IL-13) and related transcription factors (NF-κB, GATA-3, and STAT-6) (Park et al., 2018; Ren et al., 2019; Kamdi et al., 2021; Miao et al., 2021). The anti-inflammatory flavonoid compounds include apigenin, baicalein, baicalin, luteolin, lonicerin, tamarixetin, rutin, phloridzin, isoliguirtigentin, puerarin, and catechin (Table 1). The HAC_MI_ flavonoids are marked as modulation of immune cells, which is usually accompanied by anti-inflammatory effects, like luteolin and kurarinol A (Singh et al., 2021; Xu et al., 2021). These functions of activating immune cells to downregulate inflammation contains decreasing CD80/CD86 of dendritic cells and histamine, prostaglandin, pro-inflammatory, and cytokines of mast cells to suppress excessive inflammation. In addition, it also increases immune cells (T cell, macrophage, PMNs and Th2 cell) to activate innate immunity (Figure 4C). The HAC_RP_ flavonoids are the kinds of compounds that target cellular signaling pathways to combat bacteria. As shown in Table 1 and Figure 4C, the flavonoids of *Sophora flavescens* are potential agents in Tuberculosis (TB) infection therapy by enhancing macrophage autophagy to promote cellular survival and then reducing inflammation. (Kan et al., 2020). The Baicalein-7-O-β-D-glucuronide of *Scutellariae Radi* and Luteolin-7-O-β-D-glucuronide of *Impatiens balsamina* target MAPKs or Wnt/β-catenin pathway to inhibit the inflammation from bacterial LPS (Li et al., 2009; Kawai, 2018; Liu F. et al., 2020; Pereira et al., 2020).

## Conclusion

The antibacterial resistance crisis has led to a prolonged period of infection control in clinics. High-efficiency and novel antimicrobial drugs remain the most effective strategies for the treatment of multidrug-resistant and unknown pathogen infections (Brown and Wright, 2016; Theuretzbacher et al., 2020). It is clear that the main measures for the prevention and control of drug-resistant bacteria are to vigorously develop green and effective new antibacterial drugs and restore existing antibacterial drugs safely and stably. However, unclear mechanisms of antibacterial action is the main reason that hinders the development of drugs. Therefore, target identification of herbal products is necessary. In this review, we summarized both the direct-acting and host-acting antibacterial flavonoids derived from heat-clearing herbs and focused on the antibacterial modes. The current reports show the antibacterial effects of flavonoid compounds on MDR bacteria by both the direct-acting antibacterial mode and host-acting antibacterial mode (Table 1). It also proves that herbal flavonoids should be the better source for alternative antibiotics (Figure 1B and Figure 4). However, the focus on the discovery of antibacterial targets of flavonoids are the main topic in this review. In addition, the screening principles of lead flavonoids based on the antibacterial targets need more illustration. Hence, further studies ought to devote more attention to the multi-targets of flavonoids. Thus, we believe the crisis in antibiotic discovery will rapidly be solved though exploring more herbs in future. Altogether, the basis of this review aims to find the flavonoid lead compounds to guide the future drug modifications based on the structure and active antibacterial mechanism based on functional groups.

## Footnotes

1
http://www.carss.cn

2
https://amr-review.org/sites/default/files/160518_Final%20paper_with%20cover.pdf

3
http://mpns.kew.org/mpns-portal/?
